# White patients’ physical responses to healthcare treatments are influenced by provider race and gender

**DOI:** 10.1073/pnas.2007717119

**Published:** 2022-06-27

**Authors:** Lauren C. Howe, Emerson J. Hardebeck, Jennifer L. Eberhardt, Hazel R. Markus, Alia J. Crum

**Affiliations:** ^a^Department of Business Administration, University of Zurich, Zurich, Switzerland 8006;; ^b^Department of Psychology, Antioch University, Seattle, WA 98121;; ^c^Department of Psychology, Stanford University, Stanford, CA 94305

**Keywords:** racial bias, gender bias, stereotypes, medicine, patient–physician interactions

## Abstract

The healthcare workforce in the United States is becoming increasingly diverse, gradually shifting society away from the historical overrepresentation of White men among physicians. However, given the long-standing underrepresentation of people of color and women in the medical field, patients may still associate the concept of doctors with White men and may be physiologically less responsive to treatment administered by providers from other backgrounds. To investigate this, we varied the race and gender of the provider from which White patients received identical treatment for allergic reactions and measured patients’ improvement in response to this treatment, thus isolating how a provider’s demographic characteristics shape physical responses to healthcare. A total of 187 White patients experiencing a laboratory-induced allergic reaction interacted with a healthcare provider who applied a treatment cream and told them it would relieve their allergic reaction. Unbeknownst to the patients, the cream was inert (an unscented lotion) and interactions were completely standardized except for the provider’s race and gender. Patients were randomly assigned to interact with a provider who was a man or a woman and Asian, Black, or White. A fully blinded research assistant measured the change in the size of patients’ allergic reaction after cream administration. Results indicated that White patients showed a weaker response to the standardized treatment over time when it was administered by women or Black providers. We explore several potential explanations for these varied physiological treatment responses and discuss the implications of problematic race and gender dynamics that can endure “under the skin,” even for those who aim to be bias free.

## Significance

Rapid changes in the US physician workforce to include more women and people of color likely have a range of effects on healthcare. This study illustrates how long-standing societal representations of providers (i.e., as White and men) can undermine patients’ treatment outcomes. Even when White patients’ overt attitudes toward Black and women providers were positive, we found that they were less physiologically responsive to the treatment administered by these providers. These results illustrate how notions of race and gender can influence patients beneath the surface—literally under the skin—despite their professed intentions and even to their own detriment.

The face of medicine is changing. Women and people of color make up an increasing percentage of health care providers (1–3). In 2017, for the first time in history, women were the majority of accepted medical school applicants in the United States and the number of non-White accepted applicants rose to above 50%. Here, we ask whether this recent demographic shift in the race and gender of doctors is also shifting long-held, societally pervasive notions of what a doctor “looks like.”

**Fig. 1.**
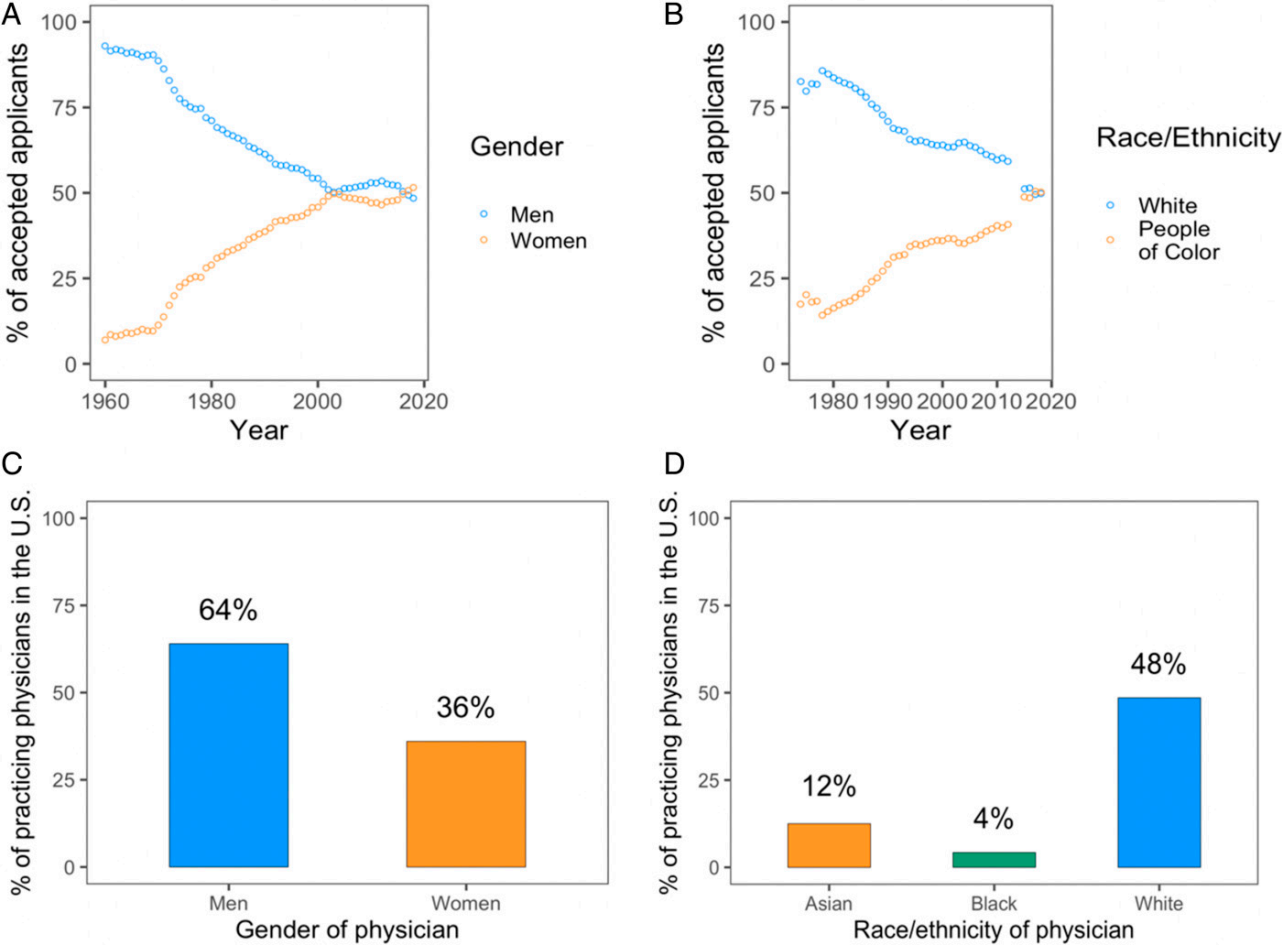
The change in the representation of women (*A*) and people color (*B*) in the number of accepted applicants to US medical schools, as well as the current representation of professionally active women physicians (*C*) and physicians of color (*D*). (*A* and *B*) From the Association of American Medical Colleges (AAMC). (*C*) From the Henry J. Kaiser Family Foundation. (*D*) From 2013 from the Association of American Medical Colleges (AAMC). AAMC data on race/ethnicity were not available for 2013 or 2014, hence explaining the gaps in the graph around these years in *B*.

Despite the increasing diversity of the medical field, for most people in most contexts, the association between “doctor” and “White man” is still likely strong and pervasive. This is hardly surprising. For most of medical history in North America, the majority of physicians fit this profile (see Fig. 1 *A* and *B*), and even now the majority of practicing physicians are still men and nearly half are White (see Fig. 1 *C* and *D*). Consequently, the emerging links between “Doctor and Woman” and between “Doctor and Black person,” for example, are likely weak. Moreover, to the extent that those associations exist, they are likely to have to compete for attention with an array of strong, frequent, and negative associations that undermine the links between women and competence and African Americans and competence (4–6).

In patient–provider interactions, as in every social encounter, people bring with them a set of learned associations about social groups that have been formed by their various life experiences (e.g., personal interactions, media exposure) (6–12). Mirroring the historical representation of doctors in actual medical practice, representations of doctors in popular media have overwhelmingly been as White men (13–15). Patients who have learned this societally pervasive “Doctor = White man” association through their actual encounters with physicians as well as through movies, television, books, and advertising may respond less positively to care from Black and women providers. These associations may exist at an implicit level even in the context of positive explicit attitudes toward Black doctors and women doctors (16, 17), and they are potentially powerful, influencing the course of medical care. Also, while it is clear from past research that being a target of bias can be harmful to health (e.g., people who face race-based discrimination face adverse physical and mental health consequences) (18), it is unclear whether viewing another social group in light of societally pervasive associations (e.g., about doctors on the basis of gender and race) can be harmful to the health of the perceiver.

Here, we focus on how the race and gender of doctors may impact patients’ responses to the expectations doctors set about medical treatment. Previous research shows that a provider’s expressed expectations for a medical treatment (i.e., that it will benefit patients) can improve patient engagement, adherence, and physiological responses to treatment (19–25). Based on these findings, we anticipate that patients who interact with a doctor whose personal characteristics (e.g., race, gender) do not conform to dominant societal representations of what a doctor looks like may be less responsive to such expectations. We hypothesize that patients may be less responsive to the exact same medical treatment when the doctor who sets expectations that this treatment will be beneficial is not a White man.

This hypothesis draws on a large and growing body of research suggesting that the total effect of a healthcare treatment depends on the social context in which that treatment takes place (25–29). The realization that the social context can influence treatment and medical outcomes is bolstered by a large body of research on the placebo effect (26). Although people may sometimes assume that actual pharmaceutical properties of a medication or treatment are solely responsible for its total benefit, placebo paradigms show that the total effect of treatment is in fact a combined product of the drug and their medical properties (e.g., acetaminophen, antihistamines), the body’s natural healing abilities (e.g., endogenous opioids and antihistamines), and the psychological and social context (e.g., what a patient believes about treatment and the qualities of the person who administers the treatment) (*SI Appendix*, Fig. S1). For example, past research suggests that a physician’s characteristics, such as their projected warmth and competence, influences how much a patient improves in response to treatment. In one recent study (22), the researchers independently manipulated whether a provider acted more or less warm, and more or less competent, toward a patient during an allergy skin prick test that induced a mild allergic reaction. The provider set positive expectations about a placebo cream (i.e., unscented hand lotion) placed on the reaction, informing patients that this cream was an antihistamine that would reduce the reaction. When the provider was both warm and competent, patients showed a stronger physiological response to the placebo treatment over time; their allergic reaction decreased the most rapidly in size, in response to the positive expectations that the provider had set. Thus, aspects of social interactions with providers can influence the degree to which the positive expectations that a provider sets about treatment ultimately influence physiological treatment response.

As in most social interactions in the United States, race and gender are likely salient aspects of the social context in patient–provider interactions (30, 31, 32). Previous research has found, for example, that patient race can influence the quality of care received from doctors in myriad ways (33–36). Here, we focus on provider race and provider gender as features of the social context that can influence patients’ response to treatment. Specifically, we ask the following: will White patients exhibit a weaker physiological response to the expectations set about treatment by doctors who are not White and men?

## Isolating the Effects of Race and Gender: Experimental Paradigm

To isolate the impact of provider race and gender on a patient’s response to treatment, we adapted a validated experimental placebo paradigm from past research (22) that standardizes most aspects of a treatment including the social context (room, situation, medical credentials) and verbal instructions (what specifically is said about the medical treatment) and randomizes patients to interact with providers of different races and genders, which are our key features of interest.

Given the complicated nature of a 6-cell study design involving actual providers, we chose to hold patient race constant, recruiting only White patients (*n* = 187) and randomizing them to interact with 1 of 13 providers who were either Asian, Black, or White and either men or women. We examined both Asian and Black doctors to compare to White doctors because this allows us to test whether differences in response to treatment are a function of broad ingroup/outgroup differences or a function of specific associations tied to one racial group.

Although Black and Asian doctors are both non-White, they are not similarly situated. First, White patients are likely less exposed to Black doctors in comparison to Asian doctors. Asian doctors are represented in greater numbers in the medical profession than Black doctors nationwide and especially in the San Francisco Bay Area, where data collection took place. In this region, representation of Asian providers is higher and in fact nearly equivalent to the number of White providers. Recent statistics about active physicians in California indicate that 27.6% identified as Asian and 32.1% identified as White, while only 2.5% identified as African American (37). Second, whereas long-held cultural stereotypes of African Americans are generally negative, Asian Americans are frequently held up as a “model minority” group (38). Third, prior research shows that affective responses to African Americans can be quite negative as well (39, 40). Given this, we would expect that patients would be more physiologically responsive to treatment from Asian doctors than African American doctors, especially among the population of patients recruited for this research.

**Fig. 2.**
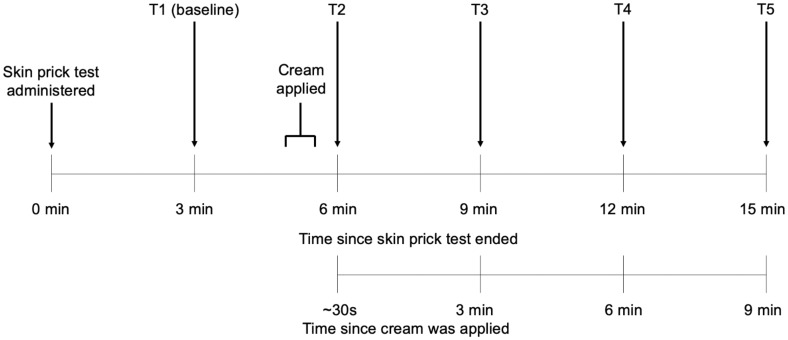
Timeline for the study skin prick procedure and measurements taken of the allergic reaction over time.

To test this, providers (blind to the study purpose and hypotheses) applied an inert cream to an induced allergic reaction as part of an allergy skin prick test. All providers set identical positive expectations about the cream (i.e., “this is an antihistamine cream that will reduce allergic reaction and itching”) to test how White patients’ physiological response to the very same treatment might differ based on the provider’s race and gender. A research assistant, blind to hypotheses, assessed how patients responded physiologically to the initial skin prick by measuring the size of their initial allergic reactions (i.e., a raised bump called the “wheal”) once, before the inert treatment was administered (*T1*, baseline 3 min post-skin prick test). The research assistant then assessed how patients responded physiologically to the inert treatment by measuring the size of the wheal four additional times over the course of several minutes after the cream was applied to its surface (*T2* = 6 min post-skin prick test and ∼30 s post-cream administration, *T3* = 9 min post-skin prick test and 3 min post-cream administration, *T4* = 12 min post-skin prick test and 6 min post-cream administration, *T5* = 15 min post-skin prick test and 9 min post-cream administration; see Fig. 2).

To understand whether there were physiological differences in the onset of allergic reactions in response to the skin prick, we first analyzed initial differences in the size of the wheal at *T1* (baseline, 3 min post-skin prick test and before the cream was applied). Then, following past research (22), we analyzed whether changes in the size of the wheal over time in response to treatment (from *T2* to *T5*) differed based on the race and gender of the provider who administered it. Comparing the rate of change in wheal size over time capitalizes on the benefits of a longitudinal design (41) and enables us to test our primary question of whether positive expectations modulated allergic reactions over time and how this effect varied based on provider race and gender. To complement this analysis, we also tested whether the size of White patients’ allergic reaction differed at *T5* (i.e., the final measurement that took place ∼9 min after the cream was applied and 15 min after the skin prick test ended), dependent on provider race and gender. To account for potential differences in initial allergic reaction size, as in past research (22), we controlled for the size of the wheal at *T1* before the cream was administered (note that the results do not substantively change with or without this covariate; *SI Appendix*). In the following sections, we examine the effect of provider gender and race on treatment response.

## Experiment Results: How Does Provider Gender Influence Physiological Reactions?

### Patient Onset of Allergic Reactions.

Provider gender did not shape the strength of White patients’ initial allergic reactions in response to the skin prick test at *T1* (*F*_ProviderGender_(1, 179) = 2.74, *P* = 0.100). Patients of women providers did show a somewhat larger initial reaction than patients of men providers; however, these results were not significant (*B* = 0.19, 95% confidence interval: [−0.04, 0.42], *SE* = 0.12, *t*(179) = 1.66, *P =* 0.100).

### Patient Response to Treatment.

**Fig. 3.**
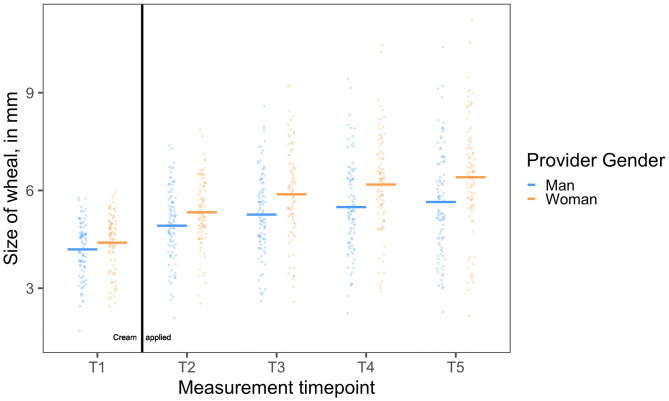
White patients responded less strongly to standardized treatment administered by a woman provider (orange points) compared to a man provider (blue points). When women providers administered a placebo cream to treat a laboratory-induced allergic reaction, White patients’ allergic reactions continued to increase at a greater rate, despite being told that the cream would reduce their reaction, than patients who received the same treatment from men providers. Scatterplots depict wheal size (in mm) by provider gender (N_Men_ = 96, N_Women_ = 91) over the time course of the study. Horizontal lines represent the mean in each group.

White patients were less responsive over time to the standardized treatment (i.e., they showed less of a decrease in allergic reaction in response to treatment) when women providers administered it, compared to men providers (*F*_ProviderGender*Time_(1, 182) = 4.50, *P* = 0.035) (see Fig. 3). Patients of women providers showed a greater increase in allergic reactions from *T2* to *T5* (i.e., less of a decrease in response to the treatment; *B*_Women__*___*SimpleEffect_ = 0.11[0.10, 0.13], *SE* = 0.01, *t*(182) = 10.55, *P <* 0.001), than patients of men providers (*B*_Men___SimpleEffect_ = 0.08[0.06, 0.10], *SE* = 0.01, *t*(182) = 7.75, *P <* 0.001).

This effect was also evident in differences in the size of White patients’ allergic reactions at the final wheal measurement. At *T5*, White patients of women providers had a larger allergic reaction size than White patients of men providers (*B* = 0.55[0.16, 0.94], *SE* = 0.20, *t*(178) = 2.73, *P =* 0.007). Thus, White patients of women providers were less physiologically responsive to treatment over time and ultimately concluded the treatment with larger allergic reactions than White patients of men providers.

## Experiment Results: How Does Provider Race Influence Physiological Reactions?

### Patient Onset of Allergic Reactions.

Unlike provider gender, provider race did shape the strength of White patients’ initial allergic reactions in response to the skin prick test at *T1* (*F*_ProviderRace_(2, 179) = 3.32, *P* = 0.038). Patients of Black providers had larger initial allergic reactions compared to patients of White providers (*B* = 0.32[0.03, 0.61], *SE* = 0.15, *t*(179) = 2.20, *P =* 0.029) and patients of Asian providers (*B* = 0.33[0.05, 0.61], *SE* = 0.14, *t*(179) = 2.32, *P =* 0.021). This is consistent with research suggesting that White Americans have more intense physiological reactions during interactions with Black Americans in certain contexts, particularly when there is high uncertainty or when expectations are violated (39, 40).

### Patient Response to Treatment.

**Fig. 4.**
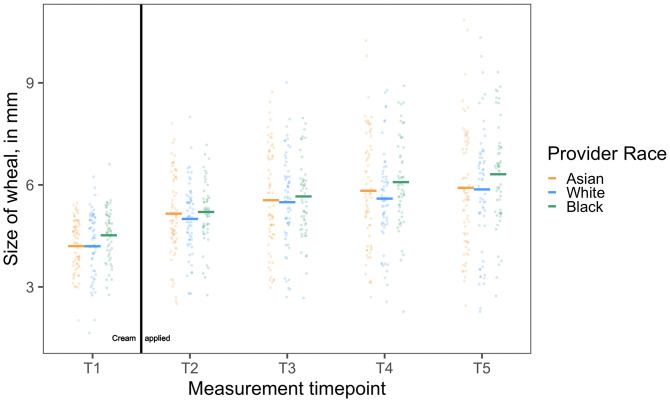
White patients responded less strongly to treatment administered by a Black provider (green points) compared to an Asian provider (orange points) or a White provider (blue points). When Black providers administered a placebo cream to treat a laboratory-induced allergic reaction, White patients’ allergic reactions continued to increase at a greater rate, despite being told that the cream would reduce their reaction, than White patients who received the same treatment from Asian or White providers. Scatterplots depict wheal size (in mm) by provider race (N_Asian_ = 70, N_Black_ = 55, N_White_ = 62) over the time course of the study. Horizontal lines represent the mean of each group.

Provider race also affected how White patients physically responded to the standardized treatment over time (*F*_ProviderRace*Time_(2, 181) = 3.40, *P* = 0.036) (Fig. 4). Patients’ allergic reactions changed differently from *T2* to *T5* in response to the standardized treatment when Black providers administered it, compared to Asian providers (*B*_Asian_Black*Time_ = 0.05[0.01, 0.08], *SE* = 0.02, *t*(181) = 2.55, *P =* 0.012). Patients of Black providers’ allergic reactions increased more from *T2* to *T5* (*B*_Black_SimpleEffect_ = 0.12[0.10, 0.15], *SE* = 0.01, *t*(181) = 9.19, *P <* 0.001) than patients of Asian providers, who showed the least increase in allergic reactions (i.e., the strongest treatment response) from *T2* to *T5* (*B*_Asian_SimpleEffect_ = 0.08[0.05, 0.10], *SE* = 0.01, *t*(181) = 6.41, *P <* 0.001). When comparing patients of Black providers to patients of White providers, the pattern was similar, although the interaction did not reach conventional significance thresholds (*B*_White_Black*Time_ = 0.03[-0.00, 0.07], *SE* = 0.02, *t*(181) = 1.84, *P =* 0.067). Patients of Black providers’ allergic reactions increased more from *T2* to *T5* (*B*_Black_SimpleEffect_ = 0.12[0.10, 0.15], *SE* = 0.01, *t*(181) = 9.19, *P <* 0.001) than patients of White providers (*B*_White_SimpleEffect_ = 0.09[0.07, 0.12], *SE* = 0.01, *t*(181) = 7.01, *P <* 0.001). Thus, compared to the patients of White and Asian providers, the patients of Black providers not only had a stronger allergic reaction to the skin prick test itself but also tended to be less physiologically responsive to the treatment over time, as their allergic reactions increased at a greater rate despite being told that the cream would reduce their reaction. White patients’ allergic reactions did not change differently from *T2* to *T5* in response to treatment administered by White providers compared to Asian providers (*B*_Asian_White*Time_ = −0.01[−0.05, 0.02], *SE* = 0.02, *t*(181) = −0.68, *P =* 0.498).

At the final wheal measurement, there were no differences evident in allergic reaction size dependent on provider race. At *T5*, White patients of Black providers had nonsignificantly larger allergic reactions compared to White patients of Asian providers (*B* = 0.14[−0.35, 0.62], *SE* = 0.25, *t*(180) = 0.55, *P =* 0.587) and compared to White patients of White providers (*B* = 0.18[−0.32, 0.67], *SE* = 0.26, *t*(180) = 0.69, *P =* 0.491). Thus, White patients of Black providers were less physiologically responsive to treatment over time, as evident in differences in the rate of change in the allergic reaction over the course of treatment, but this difference was not evident in the size of their ultimate reaction at the conclusion of treatment.^*^

## Exploratory Findings: Why Were White Patients Less Physiologically Responsive to Treatment by Black and Women Providers?

One explanation for why White patients’ physiological responses to treatment might vary by the race and gender of the provider is that the patients are biased against Black providers and women providers. At the conclusion of the study, we explored how provider race and gender might influence White patients’ responses through various bias-related mechanisms that have been documented in previous studies. We conducted exploratory analyses to test three possibilities.

First, we examine these results through the lens of the stereotype content model (4). Treatment response could be diminished because White patients judge provider warmth and competence differently and in line with the racial and gender stereotypes that are pervasive in society (4). If patients perceive Black and women providers as less warm and/or competent, this could undermine the impact of treatment expectations. Indeed, past research suggests that greater perceived provider warmth and competence enhances treatment response, increasing the potency of positive expectations (22). To test this, we measured patients’ ratings of provider warmth and competence, which they supplied at the end of their visit. Notably, however, women providers were rated as both warmer and more competent than men providers, and Black and Asian providers were rated as warmer and equally competent to White providers (*SI Appendix*), offering no evidence that patients’ explicit evaluations of these providers were guided by these negative societal stereotypes. Indeed, the White patients in this study appeared highly motivated to control biased responding. For example, on a scale measuring their internal motivation to control prejudice against racial minorities, White patients were nearly at ceiling (on a 9-point scale ranging from 1 = not all motivated to 9 = extremely motivated, participants’ modal response was 9 and their mean response was 7.7) (*SI Appendix*).

A second possibility, grounded in previous research (42, 43), is that many White patients are now unlikely to explicitly report biases, yet nevertheless hold implicit biases that undermine intergroup interactions. To test this possibility, we examined whether patients showed evidence of implicit bias through less favorable nonverbal responses or greater nonverbal anxiety toward women and Black providers. We recruited 1,213 adult participants on Amazon’s Mechanical Turk (mTurk) (71.3% White, 51.7% women, 48.1% men, 0.2% other/not reported, *M(SD)*_Age_ = 36.81(11.94)) to watch 10-s silent videoclips of the patients from the laboratory study interacting with the provider (*SI Appendix* for methodology). All videoclips cropped out the provider so that mTurk participants were unaware of the provider’s race and gender. After watching each videoclip, participants answered two questions assessing patient nonverbal bias (i.e., How much do you think the patient likes or dislikes this doctor? 1 = strongly dislikes, 7 = strongly likes; How positive or negative do you think this interaction was? 1 = very negative, 7 = very positive; *r*(6063) = 0.84, *P* < 0.001) (7) and two questions assessing perceived patient comfort (i.e., How comfortable does this patient seem? 1 = very uncomfortable, 7 = very comfortable; How anxious does the patient seem? 1 = very anxious, 7 = very calm; *r*(6063) = 0.78, *P* < 0.001).

White patients did not appear to show greater nonverbal bias when interacting with Black providers (*F*_Race_(2, 83.12) = 1.79, *P* = 0.173) (*SI Appendix*, Fig. S3). Provider race also did not predict participants’ perceptions of patient comfort (*F*_Race_(2, 81.42) = 0.97, *P* = 0.383). There was no indication of negative nonverbal bias when patients interacted with women providers either. In fact, participants rated White patients’ nonverbal reactions to women providers as more positive than their nonverbal reactions to men providers (*B* = 0.36[0.09, 0.63], *SE* = 0.14, *t*(86.6) = 2.57, *P =* 0.012) (*SI Appendix*, Fig. S4) and perceived them to be less anxious when interacting with women providers (*B* = 0.35[0.04, 0.65], *SE* = 0.16, *t*(85.0) = 2.20, *P =* 0.031). Thus, we found no evidence that subtle, negative nonverbal bias and/or greater intergroup anxiety (as indexed by observer ratings) prompted the differences in the patterns of physiological responses. Furthermore, these results held when only White mTurk participants were used in analyses (*SI Appendix*).

Finally, past research suggests that people in cross-group interactions, instead of exhibiting less favorable or more anxious nonverbal responses, may instead engage in overcompensation to actively counter bias (44, 45). Therefore, we explored whether White patients may have been more engaged in their interactions with women and providers of color. We reasoned that if White patients are exerting extra effort to be highly socially engaged with a provider (i.e., because they are motivated to avoid seeming biased toward that provider’s demographic group), this engagement may focus their attention away from the treatment and thus undermine patient responses to the expectations set by the doctor. We recruited 1,410 adult participants on mTurk (68.1% White, 52.5% women, 46.8% men, 0.7% other/not reported, *M(SD)*_Age_ = 37.40(11.94)) to view the same 10-s silent videoclips of the interactions from the laboratory study. After watching each videoclip, participants completed seven survey questions measuring how much effort the patient seemed to be investing in social engagement with the provider (e.g., How much effort is the patient making to interact with the doctor? 1 = no effort at all, 7 = a great deal of effort; How interested does the patient seem in talking to this doctor? 1 = very uninterested, 7 = very interested; *SI Appendix*, *Appendix S1* for all measures and *SI Appendix* for methodology).

**Fig. 5.**
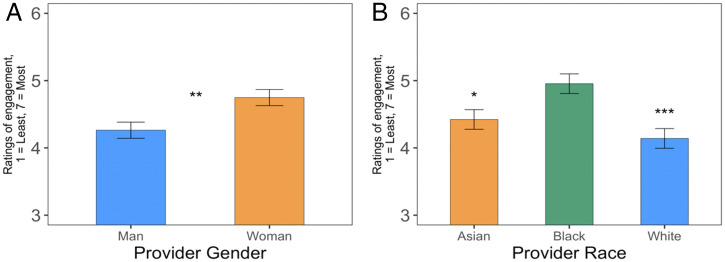
Participants perceived that White patients engaged more in interactions with women providers than men providers (*A*) and engaged more in interactions with Black providers than Asian or White providers (*B*). Means depicted are from a linear mixed-effect model predicting mTurk participants’ ratings of White patient engagement with a provider with variables indicating provider race (two dummy codes omitting White providers as the base group, N_Asian_ = 70, N_Black_ = 55, N_White_ = 62) and provider gender (one dummy code omitting men providers as the base group, N_Men_ = 96, N_Women_ = 91) as predictors. The model controlled for the patients’ perceived age and gender, as well as participant gender, and included the participant as a random intercept to account for our within-subjects design and a random intercept for each particular video to account for repeated measurements of each video across participants. Note. Error bars represent SEM. *P < 0.05, **P < 0.01, ***P < 0.001.

Results suggested that White patients in the study did, in fact, engage more with providers of color than White providers (*F*(2, 84.6) = 8.06, *P* < 0.001) (see Fig. 5*A*). Participants rated White patients as engaging more with Black providers than White providers (*B* = 0.81[0.41, 1.21], *SE* = 0.21, *t*(84.8) = 3.95 *P <* 0.001); however, they were not as inclined to rate patients as engaging more with Asian providers than White providers (*B* = 0.28[−0.11, 0.68], *SE* = 0.21, *t*(84.6) = 1.38, *P =* 0.172). Participants also rated White patients as engaging more with Black providers than Asian providers (*B* = 0.53[0.13, 0.92, *SE* = 0.20, *t*(84.4) = 2.58, *P =* 0.012). Likewise, White patients were rated to be more engaged when interacting with women providers than men providers (*F*(1, 86.6) = 8.10, *P* = 0.006) (see Fig. 5*B*); participants rated patients as more engaged in social interaction with the provider when the provider was a woman (*B* = 0.48[0.15, 0.81], *SE* = 0.17, *t*(86.6) = 2.85 *P =* 0.006). Furthermore, these results held when only White mTurk participants were used in analyses (*SI Appendix*).

## Bias under the Skin?

In patient–provider interactions, societal associations that inform who looks like a doctor and who does not not only may be harmful to the doctor but also may have some hidden costs for patients receiving treatments from women providers and providers of color. In the present study, White patients of women and Black providers, in particular, appeared less strongly influenced by the positive expectations their providers set about treatment.

We found no evidence for explicit bias in the form of ratings of competence or warmth or even in the subtle forms of bias indicated by White patients’ nonverbal behavior. Instead, the patients of women and Black providers were judged as engaging more in their interaction with these providers, not less. One possibility is that the White patients were attempting to cast aside societal associations of what a doctor looks like. Yet, their fight to push bias aside may have had an ironic effect in that it reduced the potency of their doctor’s healing words. That is, their effort to manage bias may have resulted in these White patients responding less strongly over time to the expectations those doctors were setting—that the cream they were applying would help them get better. This possibility deserves further exploration in future studies.

As anticipated, we found that White patients were less physiologically responsive to the expectations set by Black as compared to Asian providers. For a variety of reasons, a Black provider may be less consistent with the image of a doctor than an Asian provider. What we did not anticipate, however, is that White patients would respond to the expectations set by the Asian providers in the same manner as the expectations set by the White providers. White patients responded to White and Asian providers similarly from the moment their skin was pricked, to the minutes after the treatment cream was applied. It is as though Asian providers have already been absorbed into White patients’ notions of what a doctor looks like, whereas that absorption for Black doctors remains beyond reach. Future studies in different regions of the country are needed to explore this possibility further.

The study we present here was a rigorously controlled experiment in a laboratory setting in which a placebo treatment was administered and factors aside from race and gender were controlled. A benefit of this paradigm is that it shows that provider race and gender can shape treatment responses in the absence of actual pharmaceutical properties of medicine. Given that placebo effects represent common features that influence at least some portion of all treatments (*SI Appendix*, Fig. S1) (46), these effects could thus apply to a wide variety of medical contexts and treatments. Further studies that test these ideas in the administration of active medications and procedures would be valuable.

Indeed, examining the magnitude of provider race and gender effects across specific medical contexts and treatments is a ripe area for future research. For example, how might doctors’ race and gender influence patient adherence to prescribed medical regimes or to advice to schedule laboratory tests or follow-up with specialists? Or, how might race and gender influence treatment effectiveness when a patient’s health concerns are highly personal (e.g., gynecology), when there is more contact between the provider and patient (e.g., primary care), and/or when the treatment is especially risky (e.g., neurosurgery)?

Further research is also needed to understand how the race and gender dynamics observed in the current study play out over time. It could be that White patients initially show less positive responses to care from Black and women providers in the short term (e.g., responding less strongly to treatment over time as we have documented here) but that this improves in the long term over the course of interaction with these providers. It is possible, for example, that repeated exposure to a woman provider or a Black provider could lead White patients to have a stronger response to treatment. Such patients may not need to work as hard to keep bias at bay and, over time, might become increasingly attuned to the expectations these doctors set.

Contending with racial and gender inequities involves more than momentary individual effort or the desire to see those inequities disappear. What our society exposes us to across a lifetime accumulates and seeps under the skin, altering how our bodies respond and heal. Whether we want it to or not, our history of exposure leaves its trace.

## Methods

All ethical protocols, including informed consent from all participants, were followed in conducting the experiment, and approval was obtained from Stanford University’s Institutional Review Board.

We recruited 187 White patients (64.2% women, 35.3% men, *M(SD)*_Age_ = 35.06(18.82)) who consented to participate in a standardized health screening to determine their eligibility for future health behavior studies. Each patient interacted with 1 of 13 randomly assigned providers of various races and genders; 2 providers were White women, 2 were White men, 2 were Asian women, 3 were Asian men,^†^ 2 were Black women, and 2 were Black men. We selected the providers to be as similar as possible on variables other than the key variables of interest (i.e., race and gender). All providers were between the ages of 24 and 30 (*M* = 27.22, *SD* = 2.19) and were not overweight (i.e., body mass index < 25).

Providers were medical or nursing students who were blind to the study purpose and hypotheses. All providers were described to patients as medical practitioners (i.e., patients did not know whether the provider studied medicine or nursing). We standardized the behavior of providers as much as possible. Providers followed a detailed script while interacting with patients and were instructed to act in a professional manner but remain neutral with patients (e.g., minimizing small talk). Providers first asked patients about their health background, took basic health measurements (e.g., height, weight, blood pressure), and then conducted an allergy skin prick test following procedures from previous research (22). The test was conducted with histamine, to which all patients responded by experiencing itch and developing a raised bump of itchy skin (a wheal). As in past research (22), we measured how the reactions developed over the course of 15 min after the skin prick test was administered, up until ∼9 min after the expectations intervention took place. We adapted the paradigm from the previous research to allow providers to administer the procedure simultaneously to two patients, who took part in the study in separate rooms next door to one another, in order to expediate data collection efforts; the only differences between the paradigm as used in the current research and the previous study (22) is that the paradigm used in the current research included one baseline measurement (at *T1*) instead of two baseline measurements (*T1* and *T2* in the previous study) and that the intervention was administered ∼30 s or less before the *T2* measurement rather than ∼30 s or less after the *T2* measurement. Otherwise, the timing of the procedure was identical to the previous research (22).

Approximately 5 min and 30 s after the allergy skin prick test was administered, the provider placed an unscented hand lotion on the wheal of the reaction and then verbally communicated positive expectations (i.e., that the cream was an antihistamine that would reduce the reaction and itching). A research assistant who was also blind to study hypotheses and condition measured patients’ allergic reactions using a standard ruler for allergy testing at five time points throughout the examination (*T1* = 3 min post-skin prick test [baseline], *T2* = 6 min post-skin prick test and ∼30 s or less after cream administered, *T3* = 9 min post-skin prick test and 3 min postcream, *T4* = 12 min post-skin prick test and 6 min postcream, *T5* = 15 min post-skin prick test and 9 min postcream). The endpoint of the reaction was recorded by having the research assistant trace the reaction in surgical marker, lifting the tracing off the skin with transparent tape, and transferring the reaction to a paper record. See Fig. 2 for the study timeline.

### Analyses.

We used mixed-effects linear regression (package *lmer* and *lmerTest*, R version 3.3.1, https://www.R-project.org/) to examine changes in the size of patients’ allergic reaction to the skin prick test, in the 9-min period immediately after they received a placebo cream. Unstandardized regression coefficients were computed. *P* values ≤ 0.05 were considered statistically significant. As in past research (22), we focused analyses on change in wheal size and final wheal size at *T5* (the final measurement). We predicted wheal size (in mm) using provider race (two dummy codes omitting Whites as the base group, N_Asian_ = 70, N_Black_ = 55, N_White_ = 62) or gender (one dummy code omitting men as the base group, N_Men_ = 96, N_Women_ = 91) as predictors, along with their interaction with the timepoint of measurement after cream administration (∼30 s or less, 3 min, 6 min, or 9 min after the cream was applied) to measure change in wheal size over time. Data and scripts for analyses are available at https://osf.io/qcjdn/.

Analyses were conducted in the same manner as the past research using this paradigm (22). As in this past research, we controlled for wheal size directly before the cream was administered to account for individual differences in reaction size pre-cream administration, as reactions to skin prick tests can vary widely across individuals (47). Results do not substantially differ when this covariate is omitted from analyses (*SI Appendix*). In these models, we included the patient as a random intercept to account for our within-subjects design and timepoint as a random slope for each patient so we could compare the trajectory of change in wheal size across patients. As in past research (22), we controlled for patient gender in the analyses (contrast coded such that women = −1, men = 1) as well, although results do not differ when this control is omitted. To examine the effects of provider race over-and-above provider gender and vice versa, we controlled for provider race in the model examining provider gender and provider gender in the model examining provider race, although the results do not differ when these controls are omitted.

## Supplementary Material

Supplementary File

## Data Availability

Anonymized data and scripts for analyses have been deposited in the Open Science Framework at the following link: https://osf.io/qcjdn/.

## Footnotes

^*^As intersectionality is increasingly documented as an influential factor in the study of race and gender, one question is whether provider race and gender intersect to affect patient outcomes. Our study was not originally powered to pose these nuanced questions of intersectionality, but we nonetheless examined in exploratory analyses whether there was a significant three-way interaction between provider race, provider gender, and the timepoint at which patients’ wheal size was measured (i.e., *T2* to *T5*). If the interaction were significant, it would indicate that the impact of provider race on treatment response differed depending on whether the provider was a man or a woman. The three-way interaction between provider race, provider gender, and timepoint of measurement was nonsignificant, *F*(2, 178) = 0.56, *P* = 0.57. This suggests that provider race had a similar effect on treatment response for patients of both men and women providers. (*SI Appendix* for details.) However, given that our study was not powered for this intersectional analysis, these results should be interpreted with caution. Future research is needed to further probe how race and gender intersect to affect patient treatment outcomes.

^†^Providers in the study self-identified as either East Asian (hereafter referred to as Asian), African American (hereafter referred to as Black), or White American (hereafter referred to as White). One Asian man provider was unable to continue his participation in the study after data from six patients were collected, and thus, another Asian man provider was hired to collect additional data, accounting for the discrepancy in the number of Asian men providers. We include the data from patients of this provider in the analyses in this manuscript, but results do not differ when these data are omitted.
