# Supplementary material for: White patients’ physical responses to healthcare treatments are influenced by provider race and gender
Source: Proc Natl Acad Sci U S A. 2022 Jun 27;119(27):e2007717119. doi: 10.1073/pnas.2007717119 (PMC9271156; doi:10.1073/pnas.2007717119)
Supplement: Supplementary File [file pnas.2007717119.sapp.pdf]

Supporting Information

White patients' physical responses to healthcare treatments are influenced by provider race and gender

Lauren C. Howe, Emerson J. Hardebeck, Jennifer L. Eberhardt, Hazel R. Markus, and Alia J. Crum

Table of Contents

SI Figures
- Fig. S1: Components of the effectiveness of medical treatment.
- Fig. S2: Scatterplots depicting wheal size (in mm) by provider race and gender (N_{Black_Women}=27, N_{Black_Men}=28, N_{Asian_Women}=34, N_{Asian_Men}=36, N_{White_Women}=30, N_{White_Men}=32) over the timecourse of the study.
- Fig. S3: Participants did not detect more negative, non-verbal bias or greater patient discomfort when patients were interacting with providers of color as compared to White providers.
- Fig. S4. Participants rated patients as more comfortable when interacting with women providers than men providers, and they rated patients' non-verbal reactions to women providers as more positive.

SI Analyses
- Results Omitting the Control Variable of Initial Reaction Size
- Exploratory Intersectional Analyses
- Analyses Examining Ratings of Provider Warmth and Competence
  - Fig. S5: Ratings of provider competence did not differ by race, and patients rated White providers as less warm than Asian or Black providers.
  - Fig. S6: Patients rated women providers as warmer and more competent than men providers.

SI Discussion
- Exploratory Analyses: Additional Details on Methodology and Results
- Internal Motivation to Control Prejudice
  - Fig. S7: Patients' internal motivation to control prejudice in a follow-up survey.

SI References

Appendix S1: Measures used to assess patient engagement with providers

SI Figures

Fig. S1. Components of the effectiveness of medical treatment (1). People sometimes assume that actual pharmaceutical properties of a medication or treatment are solely responsible for its total benefit, but placebo paradigms show that the total effect of treatment is a combined product of the drug and its medical properties (e.g., acetaminophen, antihistamines), the body's natural healing abilities (e.g., endogenous opioids, antihistamines), and the psychological and social context (Panel A). More recently, studies have sought to isolate the contribution of elements of the psychological and social context to treatment effectiveness, including *patient mindsets* (e.g., trust, beliefs, expectations) as well as the *social context*, a broad term to describe various interlinked aspects of the clinical setting, including contextual factors (e.g., drug branding, delivery format) and interpersonal factors (e.g., provider words and actions) that can influence patient treatment outcomes through conscious or subconscious pathways (Panel B). Here we focus on how provider race and gender shape the social context of patient-provider interactions and thus may affect treatment responses (Panel C). The ratio of the placebo vs. drug effects that are responsible for the treatment effect likely differ depending on the particular treatment and context; the proportions provided in the figure are not related to the anticipated contribution size of these different factors to treatment effects.

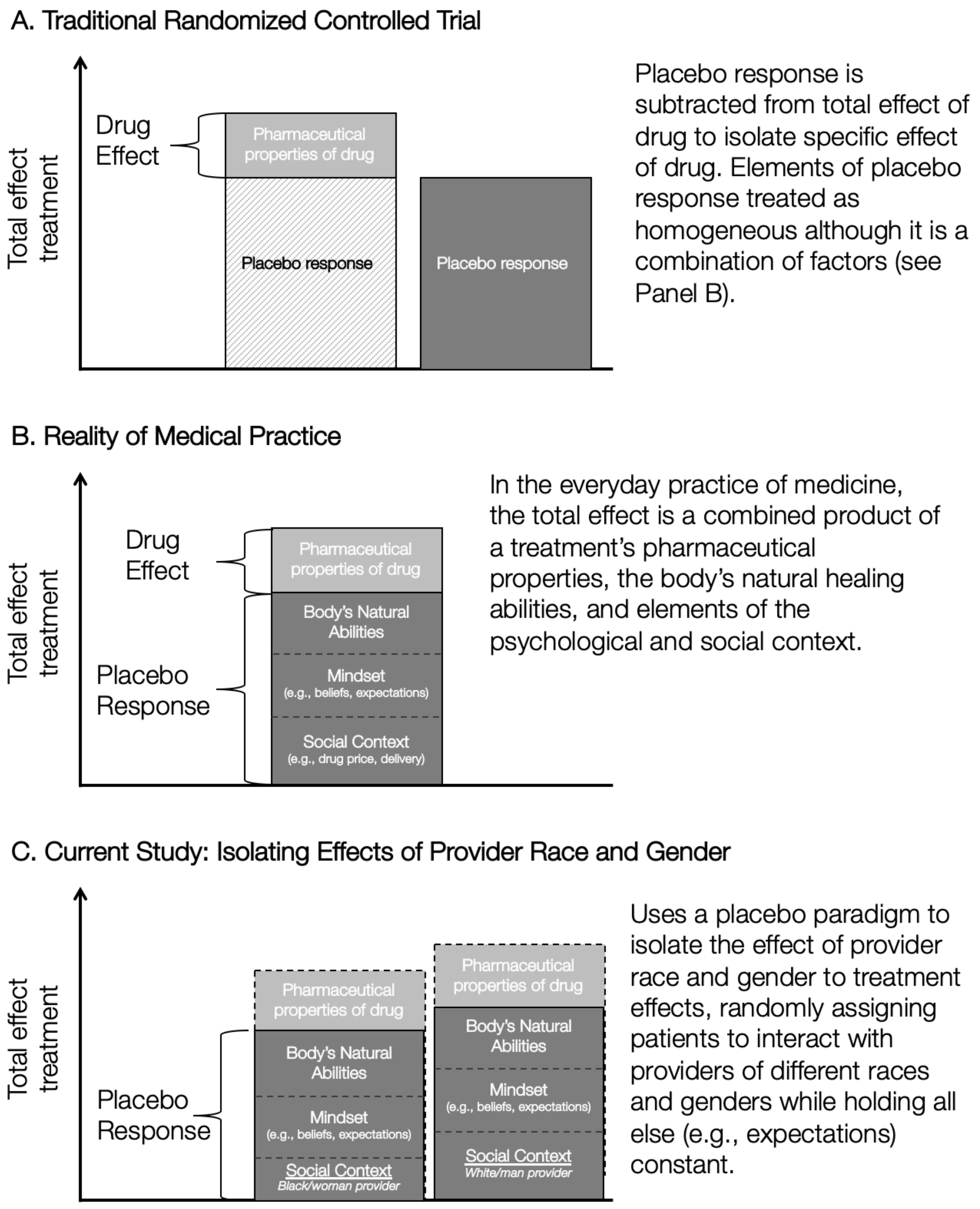
A. Traditional Randomized Controlled Trial
Total effect treatment
Drug Effect
Pharmaceutical properties of drug
Placebo response
Placebo response
Placebo response is subtracted from total effect of drug to isolate specific effect of drug. Elements of placebo response treated as homogeneous although it is a combination of factors (see Panel B).
B. Reality of Medical Practice
Total effect treatment
Drug Effect
Placebo Response
Pharmaceutical properties of drug
Body's Natural Abilities
Mindset
(e.g., beliefs, expectations)
Social Context
(e.g., drug price, delivery)
In the everyday practice of medicine, the total effect is a combined product of a treatment's pharmaceutical properties, the body's natural healing abilities, and elements of the psychological and social context.
C. Current Study: Isolating Effects of Provider Race and Gender
Total effect treatment
Placebo Response
Pharmaceutical properties of drug
Body's Natural Abilities
Mindset
(e.g., beliefs, expectations)
Social Context
Black/woman provider
Pharmaceutical properties of drug
Body's Natural Abilities
Mindset
(e.g., beliefs, expectations)
Social Context
White/man provider
Uses a placebo paradigm to isolate the effect of provider race and gender to treatment effects, randomly assigning patients to interact with providers of different races and genders while holding all else (e.g., expectations) constant.

Fig. S2: Scatterplots depicting wheal size (in mm) by provider race and gender (N_{Black_Women}=27, N_{Black_Men}=28, N_{Asian_Women}=34, N_{Asian_Men}=36, N_{White_Women}=30, N_{White_Men}=32) over the timecourse of the study. Horizontal solid lines represent the mean of each group.

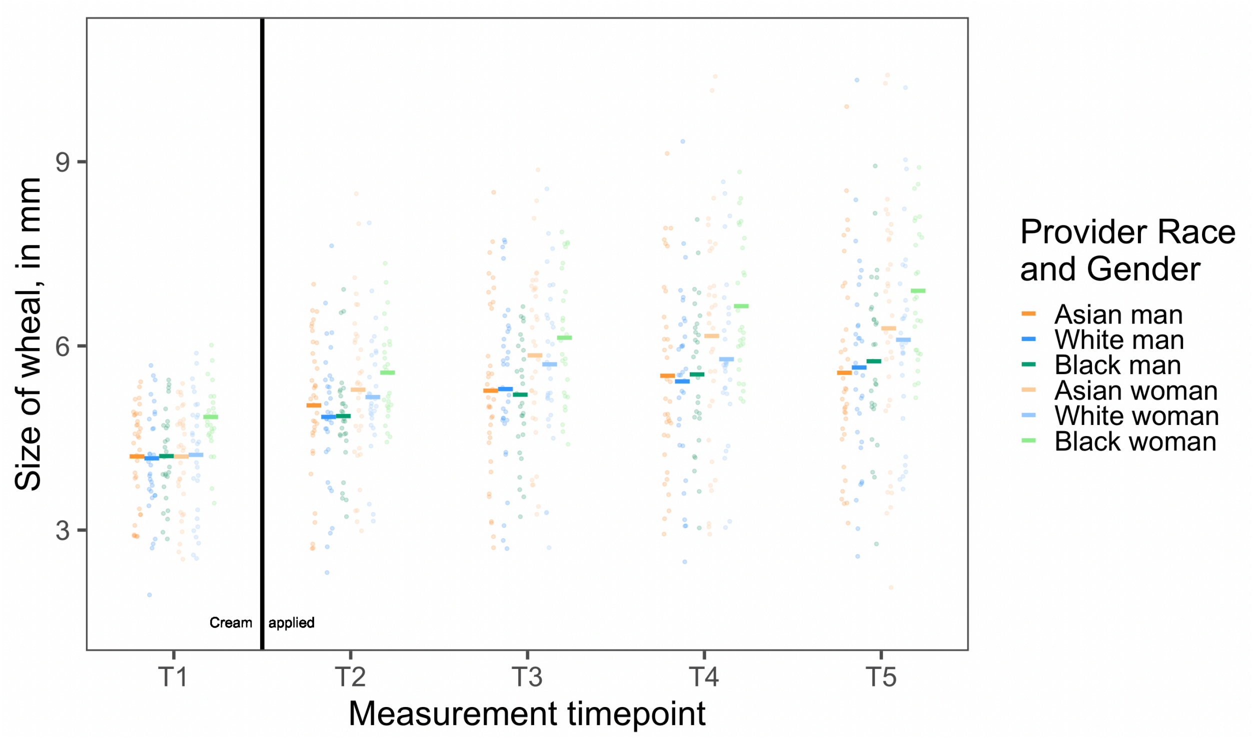

Fig. S3. Participants did not detect more negative, non-verbal bias or patient discomfort when patients were interacting with providers of color as compared to White providers.

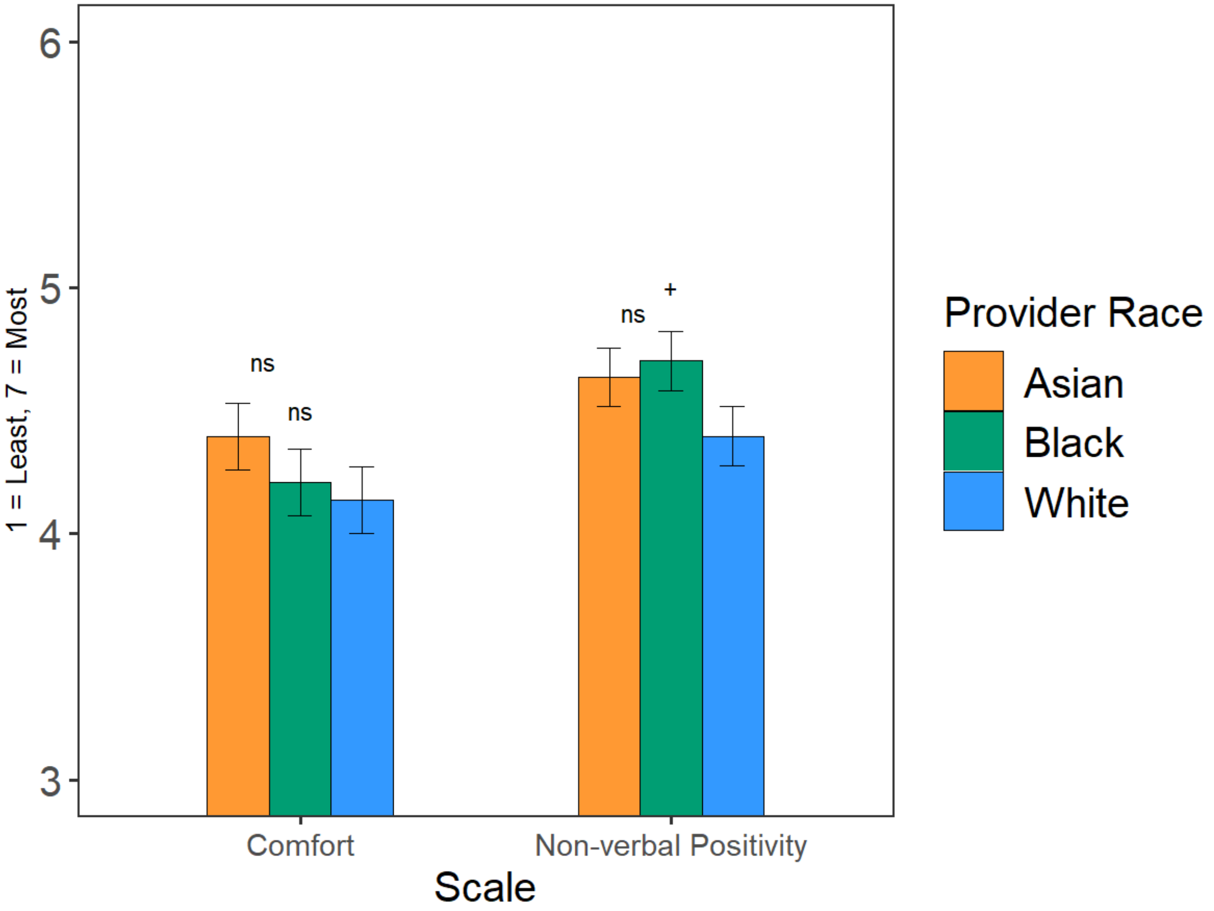

Note. Error bars represent standard error of the mean. $^{ns}p>0.10$, $^{+}p<0.10$. Participants perceived patients' non-verbal reactions to Black providers as marginally significantly more positive than non-verbal reactions to White providers, *B*=0.31[-0.02, 0.63], *SE*=0.17, *t*(83.5)=1.81, *p*=0.074, and there was a trend such that participants also perceived patients' non-verbal reactions to Asian providers as more positive than patients' non-verbal reactions to White providers, *B*=0.24[-0.09, 0.56], *SE*=0.17, *t*(83.0)=1.42, *p*=0.159. Participants did not perceive patients' non-verbal reactions to Black providers any differently from patients' non-verbal reactions to Asian providers, *B*=0.07[-0.26, 0.39], *SE*=0.17, *t*(82.8)=0.69, *p*=0.69. If anything, participants perceived patients as more comfortable when they were interacting with an Asian provider compared to a White provider, *B*=0.26[-0.11, 0.63], *SE*=0.19, *t*(81.3)=1.37, *p*=0.176, and did not perceive patients' comfort any differently when they were interacting with a Black provider compared to a White provider, *B*=0.07[-0.30, 0.44], *SE*=0.19, *t*(81.8)=0.37, *p*=0.71. Participants did not perceive patient comfort any differently when patients were interacting with a Black provider compared to an Asian provider, *B*=-0.19[-0.56, 0.18], *SE*=0.19, *t*(81.2)=-0.99, *p*=0.32.

Fig. S4. Participants rated patients as more comfortable when interacting with women providers than men providers, and they rated patients' non-verbal reactions to women providers as more positive.

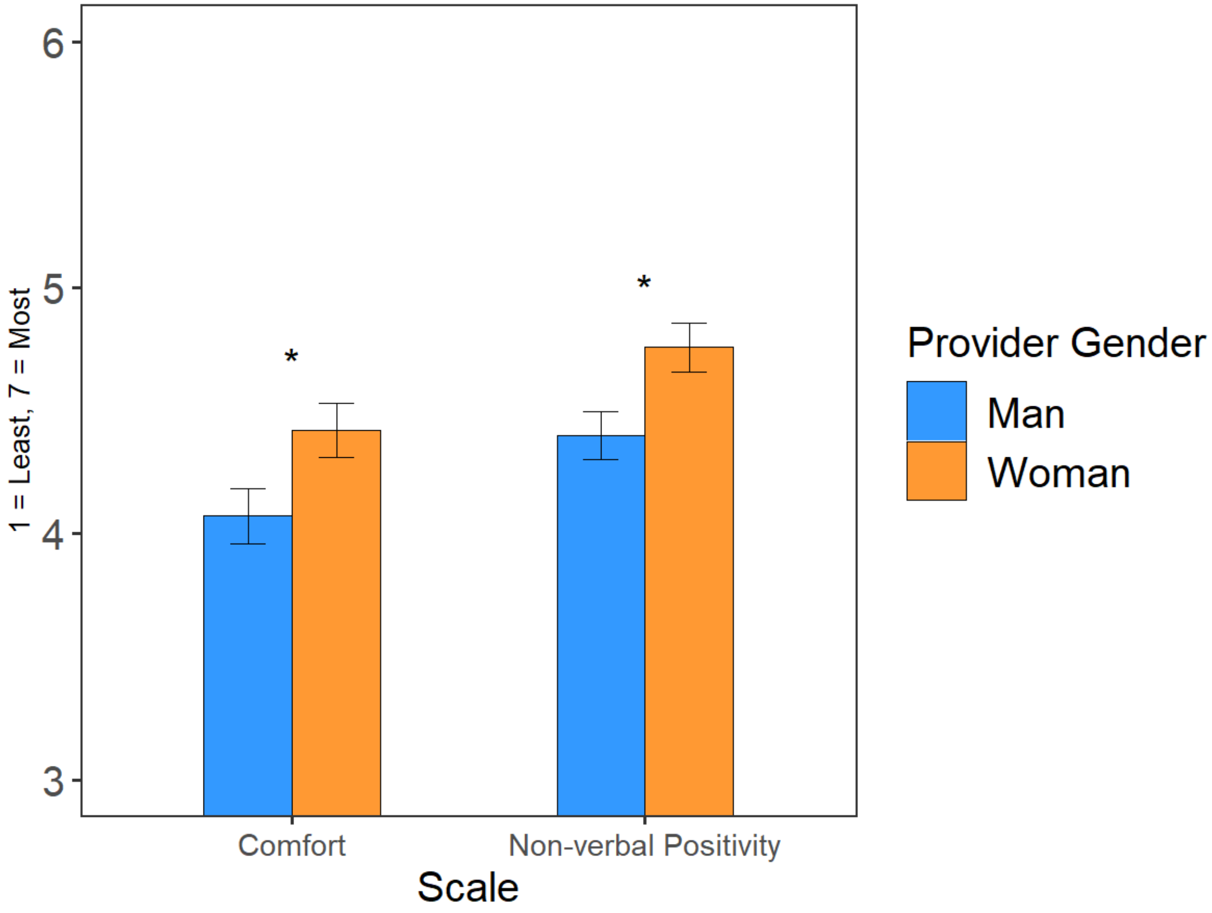

Note. Error bars represent standard error of the mean. *p<0.05

SI Analyses

Results Omitting the Control Variable of Initial Reaction Size

The analyses in the main manuscript controlled for initial wheal size in response to the skin prick test before the inert treatment was administered. This was done because research suggests that responses to skin prick tests can vary widely across individuals (2). However, the results in the main manuscript hold when this control is omitted. Results of analyses conducted in the same way as those in the main manuscript but omitting the control variable of initial reaction size remained significant and in the same direction as those reported in the main manuscript. These analyses conducted without the control variable of initial reaction size are reported below.

White patients were less responsive to the standardized treatment when women providers administered it, compared to men providers, $F_{ProviderGender*Time}(1, 184)=4.71$, $p=0.031$. Patients of women providers showed a greater increase in allergic reactions from *T2* to *T5*, $B=0.11[0.09, 0.13]$, $SE=0.01$, $t(184)=10.61$, $p<0.001$, than patients of men providers, $B=0.08[0.06, 0.10]$, $SE=0.01$, $t(184)=7.83$, $p<0.001$. At *T5*, White patients of women providers had a larger allergic reaction size than White patients of men providers, $B=0.73[0.27, 1.19]$, $SE=0.23$, $t(179)=3.11$, $p=0.002$.

Provider race also affected how White patients physically responded to the standardized treatment, $F_{ProviderRace*Time}(2, 183)=3.51$, $p=0.032$. Patients' allergic reactions changed differently from *T2* to *T5* in response to the standardized treatment when Black providers administered it, compared to Asian providers, $B_{Asian\_Black*Time}=0.05[0.01, 0.08]$, $SE=0.02$, $t(183)=2.59$, $p=0.010$, though the interaction did not reach conventional significance thresholds when compared to White providers, $B_{White\_Black*Time}=0.03[-0.00, 0.07]$, $SE=0.02$, $t(183)=1.87$, $p=0.063$. Patients of Black providers' allergic reactions increased more from *T2* to *T5*, $B_{Black\_SimpleEffect}=0.12[0.10, 0.15]$, $SE=0.01$, $t(183)=9.24$, $p<0.001$, than patients of Asian providers, who showed the least increase in allergic reactions from *T2* to *T5*, $B_{Asian\_SimpleEffect}=0.08[0.05, 0.10]$, $SE=0.01$, $t(183)=6.46$, $p<0.001$, and increased more than patients of White providers, $B_{White\_SimpleEffect}=0.09[0.07, 0.12]$, $SE=0.01$, $t(183)=7.08$, $p<0.001$. White patients' allergic reactions did not change differently from *T2* to *T5* in response to treatment administered by Asian providers compared to White providers, $B_{Asian\_White*Time}=-0.01[-0.05, 0.02]$, $SE=0.02$, $t(183)=-0.70$, $p=0.487$. At *T5*, White patients of Black providers had non-significantly larger allergic reactions compared to White patients of Asian providers, $B=0.45[-0.35, 0.62]$, $SE=0.29$, $t(181)=1.54$, $p=0.124$, and compared to White patients of White providers, $B=0.48[-0.32, 0.67]$, $SE=0.30$, $t(181)=1.60$, $p=0.112$.

Exploratory Intersectional Analyses

Do provider race and gender intersect to affect patient outcomes? Our study was not originally powered to pose nuanced questions of intersectionality, but we nonetheless examined this question in exploratory analyses. Specifically, we examined whether there was a significant three-way interaction between provider race, provider gender, and the timepoint at which patients' wheal size was measured (i.e., *T2* to *T5*). If the interaction were significant, it would indicate that the impact of provider race on treatment response differed depending on whether the provider was a man or a woman. The three-way interaction between provider race, provider gender, and timepoint of measurement was non-significant, $F(2, 178)=0.56$, $p=0.57$. This suggests that provider race had a similar effect on treatment response for patients of both men and women providers.

Given that the three-way interaction was non-significant, the lower-order interactions should be interpreted with caution; for the sake of thoroughness, however, we examined patterns among men and women providers separately. Breaking down the non-significant three-way interaction, among women providers, there was a significant two-way interaction when comparing Black women providers to White women providers, $B=0.05[0.003, 0.11]$, $SE=0.03$, $t(178)=2.05$, $p=0.042$. Patients of Black women providers' allergic reactions increased more from *T2* to *T5*, $B_{BlackWomen\_SimpleEffect}=0.15[0.11, 0.19]$, $SE=0.02$, $t(178)=7.82$, $p<0.001$, than patients of White women providers, $B_{WhiteWomen\_SimpleEffect}=0.10[0.06, 0.13]$, $SE=0.02$, $t(178)=5.26$, $p<0.001$. There was also a significant two-way interaction when comparing Black women providers to Asian women providers, $B=0.05[0.004, 0.10]$, $SE=0.03$, $t(178)=2.09$, $p=0.038$. Patients of Black women providers' allergic reactions increased more from *T2* to *T5*, $B_{BlackWomen\_SimpleEffect}=0.15[0.11, 0.19]$, $SE=0.02$, $t(178)=7.82$, $p<0.001$, than patients of Asian women providers, $B_{AsianWomen\_SimpleEffect}=0.10[0.06, 0.13]$, $SE=0.02$, $t(178)=5.52$, $p<0.001$. The two-way interaction comparing Asian women providers to White women providers was not significant, $B=0.00[-0.05, 0.05]$, $SE=0.03$, $t(178)=0.00$, $p=0.997$.

Among men providers, the two-way interaction comparing Black men providers to White men providers was not significant, $B=0.02[-0.04, 0.07]$, $SE=0.03$, $t(178)=0.59$, $p=0.557$, nor was the two-way interaction comparing Asian men providers to White men providers, $B=-0.02[-0.07, 0.02]$, $SE=0.02$, $t(178)=-0.95$, $p=0.344$. There was also not a significant two-way interaction when comparing Black men providers to Asian men providers, $B=0.04[-0.01, 0.09]$, $SE=0.03$, $t(178)=1.53$, $p=0.128$. However, the pattern was such that patients of Black men providers' allergic reactions increased the most from *T2* to *T5*, $B_{BlackMen\_SimpleEffect}=0.10[0.06, 0.14]$, $SE=0.02$, $t(178)=5.30$, $p<0.001$, followed by patients of White men providers, $B_{WhiteMen\_SimpleEffect}=0.08[0.05, 0.12]$, $SE=0.02$, $t(178)=4.73$, $p<0.001$, and Asian men providers, $B_{AsianMen\_SimpleEffect}=0.06[0.03, 0.09]$, $SE=0.02$, $t(178)=3.64$, $p<0.001$.

Taken together, these results suggest that although responsiveness to treatment from Black providers may have been weakest when those providers were also women, the pattern of results was similar for Black men providers. Both patients of Black women and Black men providers tended to respond less strongly to treatment over time relative to White and Asian providers (see Fig. S2).

Given research showing that gender concordance can impact the outcomes of patient-provider interactions, one might predict that patient gender could influence the results reported in the main manuscript in important ways (e.g., women patients interacting with a woman provider may be more responsive to the positive expectations set by these providers). We tested this possibility by conducting analyses that included an interaction between provider gender and patient gender. We also conducted analyses that included an interaction between provider race and patient gender, although we did not predict any differential responses to providers of different races/ethnicities based on patient gender. These analyses indicated that patient gender did not influence the results in meaningful ways. Below we describe the results of these analyses.

Patient gender did not interact with provider gender or provider race to impact wheal change from *T2* to *T5*, absolute value of all t's<0.97, all p's>0.34 (i.e., all of the three-way interactions between provider characteristics, patient gender, and timepoint of wheal measurement were non-significant), thus indicating that men and women patients responded similarly to men and women providers, and that gender concordance did not affect results.

Given that the study was not originally powered to detect these nuanced questions of intersectionality and concordance, these results should be considered with caution. Future research is needed to further probe how provider race and gender, as well as patient race and gender, intersect to affect patient treatment outcomes.

Analyses Examining Ratings of Provider Warmth and Competence

Ratings of provider warmth and competence. We used provider race and gender as predictors and the same dummy codes as the analyses conducted on physiological data (i.e., change in wheal size from *T2* to *T5*). As with the analyses of the physiological data, we controlled for patient gender in the analyses, though results do not differ when this control is omitted. Past literature on societal stereotypes about groups' warmth and competence (4), in light of research showing that provider warmth and competence can enhance placebo response (5), suggest that patients may respond less to treatment expectations set by Black and women providers because they perceive these providers as less warm and/or competent. Post-visit, patients rated provider warmth (7 items, α=.89, e.g., "The medical practitioner was friendly," "…made me feel at ease," and competence (11 items, α=.92, e.g., "The medical practitioner was intelligent", "…was skilled at the medical procedures") on 7-point scales (1 = *Strongly Disagree*, 7 = *Strongly Agree*) based on previous research (Howe, Goyer, & Crum, 2017) that were averaged.

Provider race indeed affected warmth ratings, $F(2,181)=8.98$, $p<0.001$ (see Fig. S5). However, patients rated Asian providers as warmer than White providers, $B=0.63[0.31, 0.95]$, $SE=0.16$, $t(181)=3.90$, $p<0.001$. Patients also rated Black providers as warmer than White providers, $B=0.58[0.24, 0.91]$, $SE=0.17$, $t(181)=3.37$, $p<0.001$. Patients did not rate Black providers any differently from Asian providers on warmth, $B=-0.05[-0.38, 0.28]$, $SE=0.17$, $t(181)=-0.32$, $p=0.75$. And, contrary to the prediction that patients might perceive Black providers as less competent, provider race did not affect ratings of competence, $F(2,181)=1.50$, $p=0.227$.

Fig. S5. Ratings of provider competence did not differ by race, and patients rated White providers as less warm than Asian or Black providers.

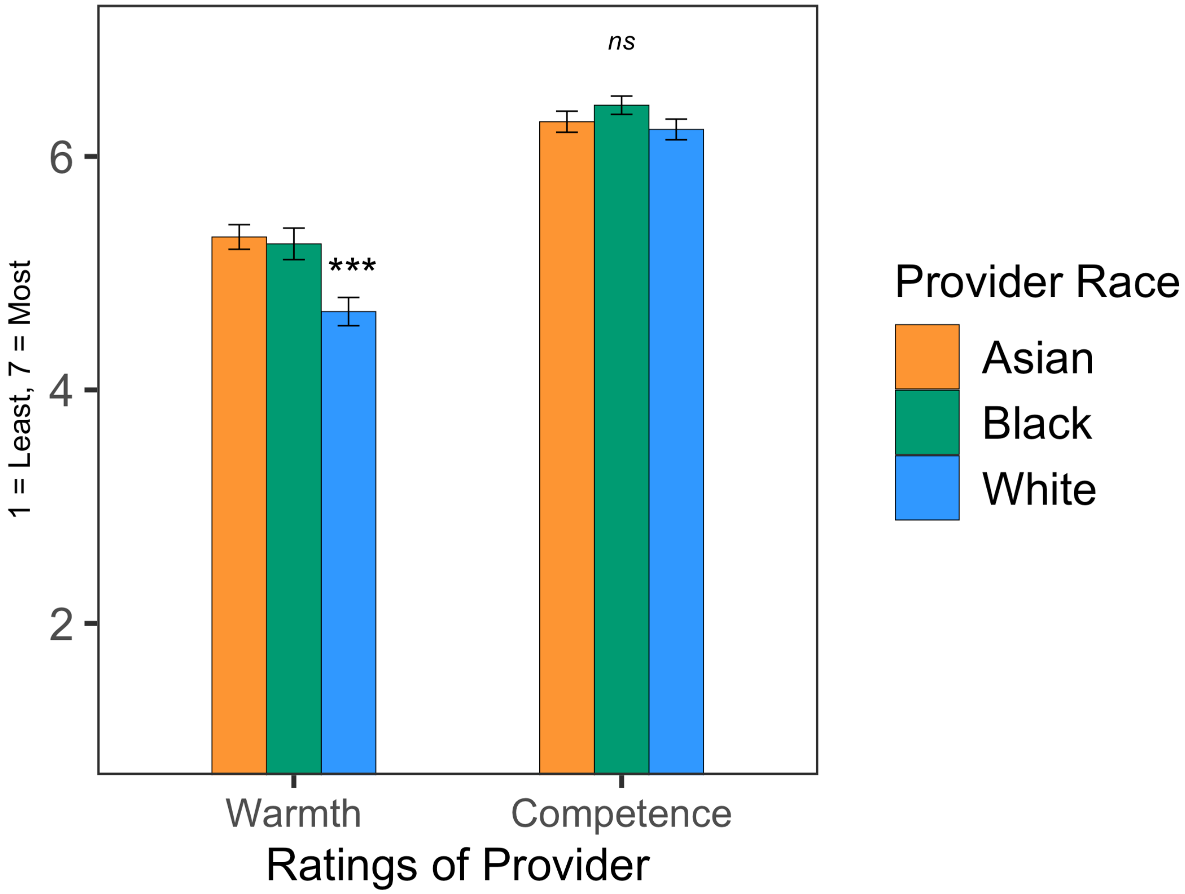

Note. Error bars represent the standard error of the mean. ***p<0.001, ^{ns}p>0.10

Provider gender also affected warmth ratings, $F(1,181)=9.57$, $p=0.002$, and competence ratings, $F(1,181)=11.51$, $p<0.001$ (see Fig. S6). Patients rated women providers as warmer than men providers, $B=0.42[0.15, 0.69]$, $SE=0.14$, $t(181)=3.09$, $p=0.002$. Patients also rated women providers as more competent than men providers, $B=0.33[0.14, 0.53]$, $SE=0.10$, $t(181)=3.39$, $p<0.001$.

Fig. S6. Patients rated women providers as warmer and more competent than men providers.

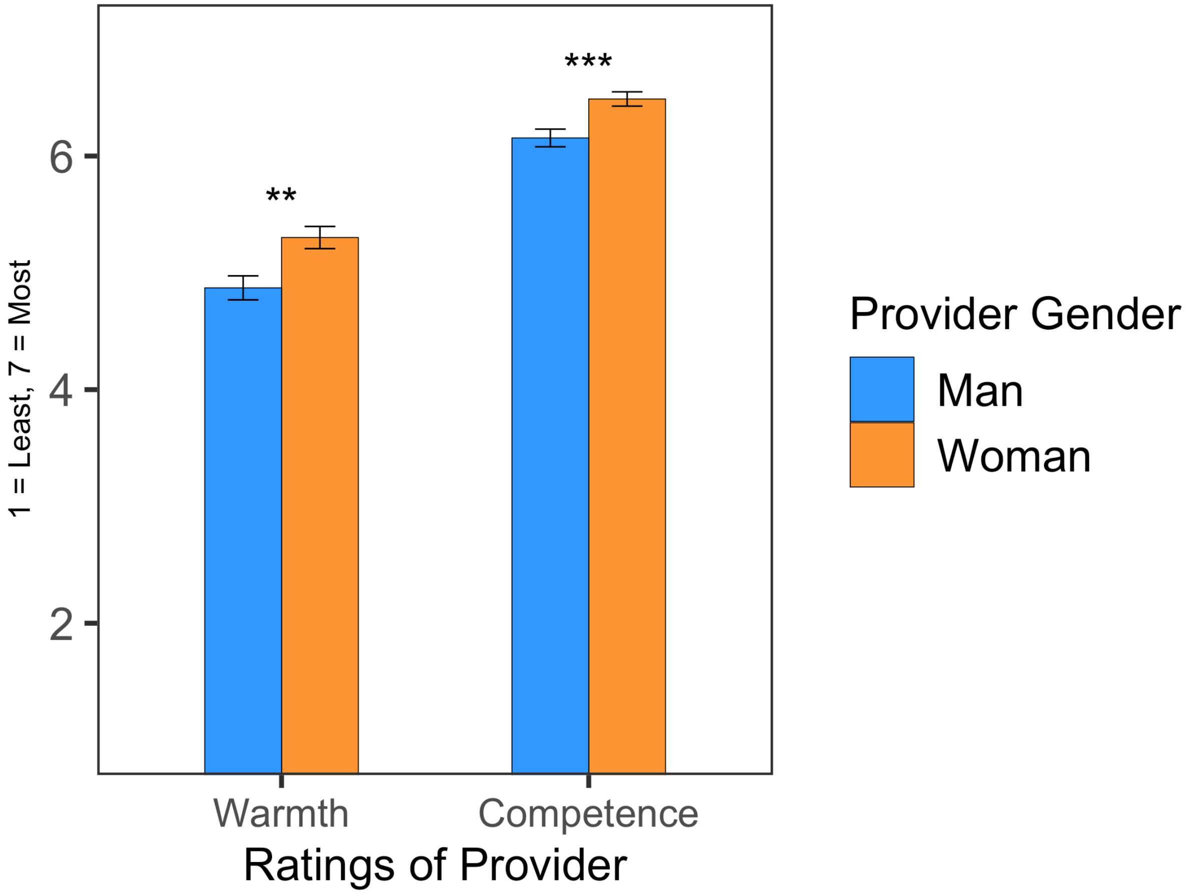

Note. Error bars represent the standard error of the mean. ***p<0.001, **p<0.01

Provider recommendations. Patients reported how likely they would be to recommend the provider to a close friend or loved one (scale points: *Definitely no, Probably no*, *Maybe*, *Probably yes*, *Definitely yes*), and we dichotomized this variable to indicate whether participants said that they would at least probably recommend the provider. We conducted chi-square tests of independence to determine how provider race and gender affected recommendations. Results are similar when recommendations are analyzed as a continuous dependent variable using linear regression.

Data regarding patients' willingness to recommend these providers to others supported these more positive perceptions of providers of color and women providers. Patients were more likely to recommend the provider if she was a woman (64 out of 91 participants, 70.3%) than if he was a man (50 out of 96 participants, 52.1%), $\chi^2(1)=6.54$, $p=0.011$. Provider race did not predict recommendations, $\chi^2(2)=3.41$, $p=0.182$, but indicated that if anything, patients were more willing to recommend providers of color (for Black providers, 36 out of 55 participants, 65.5%; for Asian providers, 46 out of 70 participants, 65.7%) than White providers (32 out of 62 participants, 51.6%).

Patients also reported how likely they would be to recommend the provider to a stranger (scale points: *Definitely no, Probably no*, *Maybe*, *Probably yes*, *Definitely yes*). We dichotomized this variable to indicate whether participants said that they would at

least probably recommend the provider. Chi-square analyses indicated that participants were more likely to recommend the provider if she was a woman (67 out of 91 participants, 73.6%) than if he was a man (55 out of 96 participants, 57.3%), $\chi^2(1)=5.50$, $p=0.019$. A chi-square based on provider race was non-significant, $\chi^2(2)=0.55$, $p=0.758$; participants were as equally willing to recommend providers of color (for Black providers, 35 out of 55 participants, 63.6%; for Asian providers, 48 out of 70 participants, 68.6%) as White providers (39 out of 62 participants, 62.9%).

In sum, patients rated Black and Asian providers as more warm than White providers, and women providers as both warmer and more competent than men providers. In addition, patients were more likely to recommend women providers over men and were just as likely to recommend providers of color as White providers. Thus, patients' ratings of provider warmth and competence do not explain why patients responded less to the expectations set by Black and women providers. There is an intriguing disconnect between patients' self-reported perceptions of the providers and their actual physical response to the providers' treatment, aligning with literature suggesting that people often do not report explicitly biased attitudes but may show evidence of bias in other ways (3).

SI Discussion

Exploratory Analyses: Additional Details on Methodology and Results

Below, we include additional details about the methodology used in the exploratory analyses presented in the discussion section. We also include supplemental analyses involving only White participants on Amazon's Mechanical Turk (mTurk).

Patient non-verbal bias and anxiety. First, we created a pool of 90 videoclips of patients interacting with the healthcare providers during the experiment. Each participant watched a unique, randomly selected subset of six videoclips. Each participant watched one videoclip of a patient (i.e., the participants from the lab study) interacting with a healthcare provider of each race and gender (i.e., one Asian man, one Asian woman, one Black man, one Black woman, one White man, one White woman). Each videoclip was approximately 10 seconds long and silent and the researchers cropped out the healthcare provider so that participants were unaware of the healthcare provider's race and gender. Participants thus only saw silent videoclips of the patient's non-verbal behavior. Participants also reported the gender of the patient in the video and estimated the age of the patient in the video, as these factors might also affect interaction quality with providers.

The R package *rmcorr* was used to calculate repeated measures correlation coefficients, thus taking into account the fact that there were six measurements of these variables per participant. We used mixed-effects linear regression to predict ratings of comfort/anxiety and patient engagement with providers. We included race (two dummy codes omitting Whites as the base group) and gender (one dummy code omitting men as the base group) as predictors. We included random intercepts for participant and target (i.e., the particular patient that participants evaluated) to account for correlated responses across participants and across targets. We controlled for the gender and perceived age of the patient in the video (male/female) as well as the gender of the participant in the analyses; results are not affected if these controls are removed.

As a secondary prediction, we predicted that participants would rate patients as more uncomfortable in the clips from right after when the skin prick test was conducted than in the other two clips (initial entry, before skin prick test). This is because the skin prick test involved patients having their skin touched by providers, and we thought that this physical contact might enhance discomfort in cross-race interactions. Accordingly, the clips were taken either during the initial interaction (i.e., right as the provider entered the door of the exam room), right before skin prick test, or right after skin prick test. The timing of the clip was randomized across participants. We tested whether the timing of the interaction (initial entry of provider, before skin prick test, after skin prick test) moderated the effects, by including an interaction between this variable (dummy coded to omit clips that were filmed after the skin prick test) and provider race/gender as a fixed effect in our model. Clip timing did not moderate the effects, absolute value of all t's<1.50, all p's>0.139, and thus we do not discuss this variable further.

When analyses included only White participants on MTurk, they were similar to the analyses presented in the main manuscript. Patients did not appear to show greater non-verbal bias when interacting with Black providers, $F(2, 83.22)=1.50$, $p=0.229$. Provider race also did not predict participants' ratings of patient comfort, $F(2, 81.42)=0.73$, $p=0.484$. There was no indication of negative non-verbal bias when

patients interacted with women providers either. In fact, participants rated patients' non-verbal reactions to women providers as more positive than their non-verbal reactions to men providers, B=0.37[0.10, 0.64], SE=0.14, t(86.9)=2.62, p=0.010 and perceived them to be less anxious when interacting with women providers, B=0.36[0.05, 0.66], SE=0.16, t(85.1)=2.24, p=0.028.

Patient engagement with providers. All of the measures of patient engagement in Appendix S1 correlated highly with one another (r's>0.60) except for one ("How preoccupied does the patient seem by interacting with this doctor?", r's<0.12). This question was thus omitted and all others were combined into a composite scale measuring social engagement with the providers (6 items, alpha = 0.95). Results are similar when each of these measures is examined separately.

When analyses included only White participants on MTurk, they were similar to the analyses presented in the main manuscript. Results suggested that White patients in the study did, in fact, *engage more* with providers of color than White providers, F(2, 84.6)=8.34, p<0.001. Participants rated patients as engaging more with Black providers than White providers, B=0.83[0.43, 1.23, SE=0.21, t(84.9)=4.02, p<0.001, however, they were not as inclined to rate patients as engaging more with Asian providers than White providers, B=0.29[-0.11, 0.68], SE=0.21, t(84.5)=1.439, p=0.167. Participants also rated White patients as engaging more with Black providers than Asian providers, B=0.54[0.15, 0.94], SE=0.21, t(84.4)=2.64, p=0.010. Likewise, White patients were rated to be more engaged when interacting with women providers than men providers, F(1, 87.1)=10.03, p=0.002; participants rated patients as more engaged in social interaction with the provider when their provider was a woman, B=0.54[0.21, 0.87], SE=0.17, t(87.1)=3.17, p=0.002.

Internal Motivation to Control Prejudice

In a follow-up survey conducted approximately one month after the end of the study, 140 of the White patients from the lab study completed an established measure of their internal motivation to control prejudice against racial minorities (6) (4 items, alpha = 0.76, e.g., “I attempt to act in non-prejudiced ways toward racial and ethnic minorities because it is personally important to me”, 1 = *not all motivated* to 9 = *extremely motivated*). White patients were nearly at ceiling on this measure (on the 9-point scale ranging from 1 = *not all motivated* to 9 = *extremely motivated*, participants' modal response was 9 and their mean response was 7.74). See Figure S7 below.

Fig. S7. Participants' internal motivation to control prejudice in the follow-up survey.

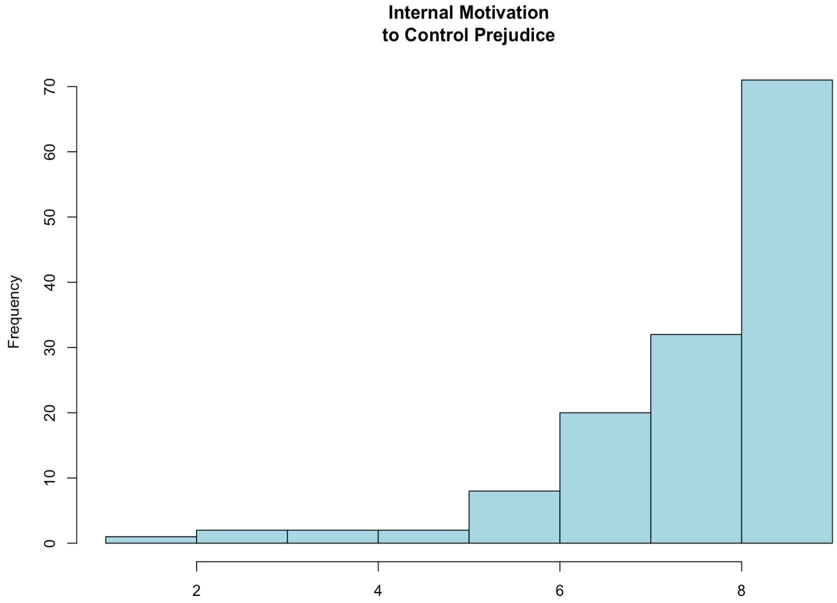

SI References

1. Crum AJ, Leibowitz KA, Verghese A (2017) Making mindsets matter. *Br Med J* 356:674–678.
2. Scherer K, Grize L, Schindler C, Surber C, Biercher AJ (2007) Reaction pattern to histamine and codeine in a human intradermal skin test model. *Clin Exp Allergy* 37:39-46.
3. Dovidio JF, Kawakami K, Johnson C, Johnson B, Howard A (1997). On the nature of prejudice: Automatic and controlled processes. *J Exp Soc Psychol* 33(5):510-540.
4. Fiske ST, Cuddy AJC, Glick P, Xu J. (2002). A model of (often mixed) stereotype content: Competence and warmth respectively follow from perceived status and competition. *J Pers Soc Psychol* 82(6):878-902.
5. Howe LC, Goyer JP, Crum AJ (2017). Harnessing the placebo effect: Exploring the influence of physician characteristics on placebo response. *Health Psychol* 36(11):1074–1082.
6. Plant EA, Devine PG (1998). Internal and external motivation to respond without prejudice. *J Pers Soc Psychol* 75(3):811-832.

Appendix S1

Measures used to assess patient engagement with providers

1. How much effort is the patient making to interact with this doctor? 1=no effort at all, 7=a great deal of effort
2. How much effort is the patient making to listen to and understand this doctor? 1=no effort at all, 7=a great deal of effort
3. How much does the patient seem to trust this doctor? 1=distrust a great deal, 7=trust a great deal
4. How interested does the patient seem in getting to know this doctor? 1=very uninterested, 7=very interested
5. How interested does the patient seem in talking to this doctor? 1=very uninterested, 7=very interested
6. How much attention does the patient seem to be paying this doctor? 1=no attention at all, 7=a great deal of attention
7. How preoccupied does the patient seem by interacting with this doctor? 1=not preoccupied at all, 7=very preoccupied*

*This item was excluded from analysis as it did not correlate well with the other items.
